# Temperature-Based Predictions for West Nile Virus Outbreaks in Endemic Regions of Continental Croatia

**DOI:** 10.3390/pathogens15050509

**Published:** 2026-05-08

**Authors:** Ljubo Barbić, Gorana Miletić, Maja Maurić Maljković, Vladimir Stevanović, Vladimir Savić, Ivona Ćorić, Maja Bogdanić, Ivana Rončević, Ana Sanković, Marko Belamarić, Tatjana Vilibić-Čavlek

**Affiliations:** 1 Department of Microbiology and Infectious Diseases with Clinic, Faculty of Veterinary Medicine, University of Zagreb, 10000 Zagreb, Croatia; gmiletic@vef.unizg.hr (G.M.); vladostevanovic@gmail.com (V.S.); icoric@vef.unizg.hr (I.Ć.); 2 Department for Animal Breeding and Livestock Production, Faculty of Veterinary Medicine, University of Zagreb, 10000 Zagreb, Croatia; mmauric@vef.unizg.hr; 3 Poultry Center, Croatian Veterinary Institute, 10000 Zagreb, Croatia; v_savic@veinst.hr (V.S.); ironcevic@veinst.hr (I.R.); 4 Department of Virology, Croatian Institute of Public Health, 10000 Zagreb, Croatia; maja.bogdanic@hzjz.hr; 5 School of Medicine, University of Zagreb, 10000 Zagreb, Croatia; 6 Department of Microbiology, University of Applied Health Sciences, 10000 Zagreb, Croatia; asankovic@zvu.hr; 7 Department of Epidemiology, Croatian Institute of Public Health, 10000 Zagreb, Croatia; mbelamaric123@gmail.com

**Keywords:** West Nile virus, climate, seroprevalence, horses, humans, Croatia

## Abstract

Weather conditions, especially temperature, rainfall, and humidity, affect the transmission and spread of West Nile virus (WNV). This study investigated the effects of weather patterns on WNV activity in Croatia using climatological, equine seroprevalence, and human case data collected in two endemic continental Croatian regions with high WNV activity from 2015 to 2024. Overall equine WNV IgG prevalence was significantly higher in East Croatia (30.34%) than in Central Croatia (10.90%) and increased over time in both regions with a similar temporal pattern, indicating a shared upward trend in viral circulation. Higher equine seroprevalence was observed in areas with confirmed recent equine infections (IgM positive) within the same transmission season. In addition, human cases and recent equine infections were significantly associated with higher equine seroprevalence in the following season, with increases of 4% and 7%, respectively. In contrast to precipitation and humidity, temperature was significantly associated with human WNV cases, whereas no comparable effect was found in horses. Temperatures in February, April, May, and October emerged as key predictors, and the model including mean April and May temperatures showed the best predictive performance for human WNV cases, further supported by analysis of the epidemic year 2018. However, when 2018 was excluded, the effects of temperature remained significant only for May and July, with increased May temperatures emerging as the most important predictor of WNV activity in the upcoming transmission season.

## 1. Introduction

West Nile virus (WNV) is a mosquito-borne arbovirus of the family *Flaviviridae*, genus *Orthoflavivirus*, Japanese encephalitis serocomplex. In nature, WNV is maintained in an enzootic transmission cycle between birds and mosquitoes, primarily of the genus *Culex*. Occasionally, the virus spills over to humans, horses, and other mammals; however, these hosts usually develop low-level viremia and therefore do not significantly contribute to further transmission, making them incidental or dead-end hosts [1].

WNV is the most widespread mosquito-borne virus in Europe, posing a significant public health concern. The WNV activity has increased substantially over the past two decades across Europe, with both the number of cases and the geographic distribution expanding. Large outbreaks have been reported, particularly in southern, central, and eastern Europe. Most human WNV cases are reported during the summer and early autumn months, a pattern that follows the seasonal dynamics of mosquito vectors. In recent years, several European countries, including Italy [2], Greece [3], Romania [4], Hungary [5], Spain [6], and Croatia, have reported recurrent outbreaks and a noticeable increase in the incidence of WNV infection [7].

Increasing evidence demonstrates that climatic conditions influence both the transmission dynamics and geographic distribution of WNV. High temperatures, rainfall patterns, and humidity significantly impact the survival and reproduction of mosquito vectors. Temperature is one of the most important climatic factors affecting WNV transmission. Higher temperatures accelerate the developmental rate and reproductive cycle of mosquitoes, as well as shorten the extrinsic incubation period, thereby increasing the likelihood of transmission [8]. Seasonal climate patterns, therefore, affect the abundance of mosquito populations and the intensity of virus circulation among bird reservoirs. As a result, WNV outbreaks are more common during warm seasons, especially in late summer and early autumn. Climate change may further influence the geographic spread of the virus by expanding suitable habitats for mosquito vectors and altering migration patterns of birds, which are the main reservoir hosts in the natural transmission cycle [9,10,11].

In Croatia, since the first reported human cases, WNV became endemic in continental regions. The association of WNV infections and climate was observed during the largest outbreak recorded in the country in 2018. From April to October 2018, climate conditions in continental Croatia were very or extremely warm, with temperatures above long-term averages, resulting in an early start of transmission season [12]. Similarly, a study on the 2024 transmission season in Croatia reported that extremely warm climate conditions may have contributed to the intense arboviral season and increased circulation of WNV and other flaviviruses. Additionally, correlations were observed between precipitation levels and the number of arboviral infections in several Croatian counties, including Zagreb and its surrounding regions [13].

The growing geographic spread and recurring WNV outbreaks highlight the importance of integrated surveillance, vector control, and early detection systems. This study aimed to identify climatic drivers (e.g., temperature, precipitation, humidity) that influence WNV transmission dynamics in Croatia, and the predictive model for human WNV infections.

## 2. Materials and Methods

### 2.1. Climatological Data

**Figure 1:**
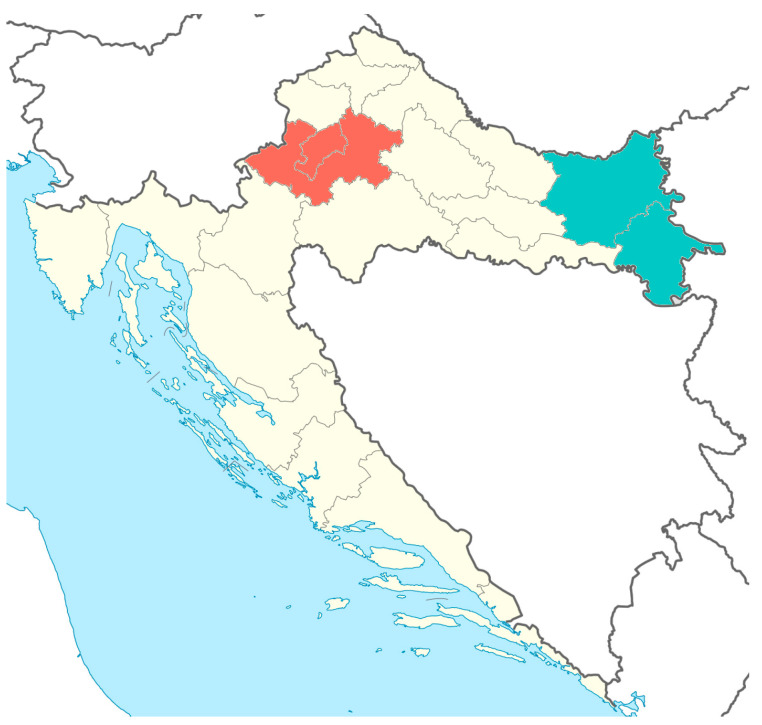
Analyzed regions in continental Croatia.

Data on temperature, precipitation, and humidity were provided by the Croatian Meteorological and Hydrological Service, which records these parameters as part of its daily activities. Data for the study period were collected at four meteorological or climatological stations in continental Croatia. Two of them are located in eastern Croatia (Osijek, representing Osijek-Baranja County, and Vukovar, representing Vukovar-Srijem County), and two in central Croatia (Zagreb, representing the City of Zagreb, and Pisarovina, representing Zagreb County) (Figure 1). These stations were selected to cover the two Croatian areas with the highest number of WNV-related human illnesses during the study period and to enable comparison of climatological indicators at the local and regional levels. Data from these stations were collected daily, and monthly values were calculated as the arithmetic mean of daily values for each month from 1 January 2015, to 31 December 2024 (temperature and humidity). Precipitation was calculated and analyzed as cumulative monthly rainfall. Climatological factors at the annual level were calculated as the arithmetic means of monthly values over the same period.

### 2.2. Data on West Nile Virus Seroprevalence in Horses and Human Cases

Serological testing of horses for WNV was conducted to determine seroprevalence (IgG antibodies) and recent infections (IgM antibodies) during the study period as part of the early detection and surveillance program for West Nile disease in the Republic of Croatia. This program is defined and implemented annually by the Veterinary and Food Safety Directorate of the Ministry of Agriculture of the Republic of Croatia. Horse serum samples submitted to the Faculty of Veterinary Medicine, University of Zagreb, for other diagnostic purposes (mainly surveillance of equine infectious anemia) were tested. Within this pool of samples, samples for WNV surveillance are selected randomly and proportionally to the horse population in each area. Previously tested horses with a positive WNV result were excluded from further WNV testing; however, negative animals from holdings that had been sampled in previous years were not excluded. Year-to-year variations in sample size were therefore attributable to changes in the scope of the national surveillance program, which was reduced from 2019 onward. Vaccination of horses against WNV was not routinely performed in Croatia throughout the study period. Therefore, the likelihood that any of the sampled animals had been vaccinated is considered negligible. Accordingly, WNV IgG seropositivity in this study was interpreted as evidence of previous natural exposure rather than vaccination-induced antibodies.

A total of 4986 samples from all four counties included in this study were tested in the period from 2015 to 2024. All samples were tested to determine WNV IgG seroprevalence using a competitive ELISA test (INgezim^®^ West Nile, Gold Standard Diagnostics, Madrid, Spain) and to confirm recent infections using an antibody capture ELISA (INgezim^®^ West Nile IgM, Gold Standard Diagnostics, Madrid, Spain), according to the manufacturer’s instructions. A virus neutralization test was not performed to confirm the positive ELISA result. However, previous studies from the same regions indicated that USUV seroprevalence was low, both in horses [14] and in other animal species [15]. Although TBEV seroprevalence was higher, cross-reactivity between WNV and TBEV in ELISA assays has been reported rarely [16]. Consequently, the possibility of flavivirus cross-reactivity was unlikely to significantly influence the WNV testing results or interpretation of temporal trends.

The Reference Center for Diagnostics and Surveillance of Viral Zoonoses of the Croatian Ministry of Health at the Croatian Institute of Public Health provided data on human WNV cases [7,13,17]. All patients were classified as confirmed WNV cases according to the criteria of the European Centre for Disease Prevention and Control (ECDC), including: (a) virus isolation; (b) detection of viral RNA in blood and/or cerebrospinal fluid (CSF); (c) a specific IgM antibody response in CSF; and/or (d) a high IgM titer and IgG antibody detection confirmed by a virus neutralization test [18].

### 2.3. Statistical Analysis

Descriptive statistics are presented as numbers and percentages. Differences between quantitative variables were assessed using the Mann–Whitney U test, and associations were evaluated using the Spearman rank correlation coefficient (ρ), due to the small sample size. Temporal trends were evaluated using linear regression models (value ~ year). In addition, Spearman rank correlation coefficients were calculated between meteorological variables and year to assess monotonic trends independent of linearity assumptions. Regional temperature trends were derived from mean annual temperature series of stations within each region. Seroprevalence was analyzed using binomial generalized linear models (GLMs), with the number of positive samples and the number tested specified as a binomial outcome. Results are presented as odds ratios (ORs) with 95% confidence intervals (CIs) based on Wald statistics. The association between climatological variables and the number of human cases and acute infections in horses was assessed using Poisson generalized linear models with a log link. Models were adjusted for year (mean-centered). To account for multiple comparisons across monthly models, *p*-values were adjusted using the Benjamini–Hochberg false discovery rate (FDR) method. Missing data for acute horse infections in 2023 were coded as NA and handled using complete-case analysis. To account for variation in sampling effort in horses, the logarithm of the number of tested horses was included as an offset. Results are presented as incidence rate ratios (IRRs) with 95% confidence intervals, estimated using robust standard errors to account for potential overdispersion. A limited set of candidate models, pre-specified based on biological plausibility and guided by initial analyses, was evaluated and compared using the Akaike Information Criterion (AIC). Furthermore, generalized additive models (GAMs) were used to assess the association between temperature and human cases, allowing for potential non-linear effects of temperature. Month-specific smooth terms were included to evaluate whether the temperature–response relationship varied across months, with adjustment for year (mean-centered). To evaluate the delayed effects of climatic conditions, lagged analyses were performed by relating temperature variables in year t to seroprevalence in year t + 1. An outbreak year was defined as a year in which the number of cases exceeded the mean plus two standard deviations of the study period. The outbreak threshold was calculated based on baseline years excluding 2018. Notably, 2018 (*n* cases =38) exceeded the threshold regardless of whether it was included (29.54) or excluded (9.76) from baseline calculations. To identify anomalous temperature patterns, monthly Z-scores were calculated for the epidemic year 2018 relative to the mean and standard deviation of the remaining study period. Values exceeding ±2 standard deviations were considered indicative of substantial deviations from the long-term average. To identify the best predictive model for human WNV cases, models were evaluated using a forward-chaining approach, where each year was predicted using only data from preceding years. Differences were considered significant at *p* < 0.05. Statistical analyses were performed using R 4.5.2 (R Core Team, Vienna, Austria, 2025).

## 3. Results

### 3.1. Climatological Factors

The mean annual temperature during the study period increased significantly in the overall study area and in both Eastern and Central Croatia (0.16 °C per year; *p* = 0.02 for all), with positive Spearman correlations also supporting an increasing temporal trend. No significant trends were observed for annual precipitation or humidity (Table 1).

### 3.2. West Nile Virus IgG Prevalence in Horses

WNV IgG antibodies were detected in 1059/4986 horse samples (21.24%, 95% CI 20.11–22.40). The overall seroprevalence was 30.34% (95% CI 28.61–32.11) in Eastern Croatia and 10.90% (95% CI 9.74–12.27) in Central Croatia, with a significant difference (χ^2^ = 277.97, *p* < 0.001; RR = 2.77, 95% CI 2.44–3.15; OR = 3.54, 95% CI 3.04–4.13). In each year of the study, WNV IgG prevalence was higher in Eastern Croatia than in Central Croatia (Table 2).

**Figure 2:**
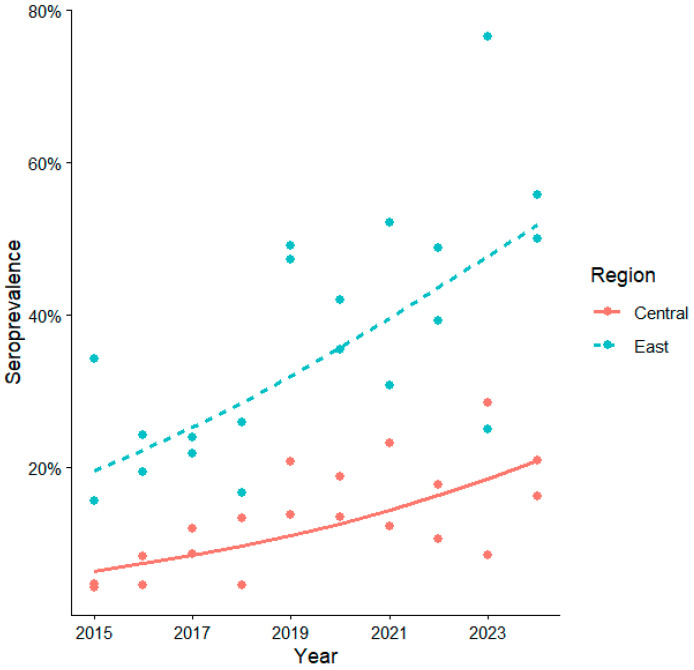
Logistic trend of WNV IgG seroprevalence in central and eastern Croatia (2015–2024).

The seroprevalence trend showed an increase in seropositivity each year (OR = 1.16, 95%CI 1.11–1.21, *p* < 0.001), with similar trends in both regions, Central (OR = 1.16, 95% CI 1.11–1.21, *p* < 0.001) and Eastern Croatia (OR = 1.18, 95% CI 1.14–1.21, *p* < 0.001). The interaction between area and year indicates no significant differences, meaning the increasing trend was similar in both regions (OR = 1.01, 95% CI = 0.96–1.07, *p* = 0.58; Figure 2).

### 3.3. Recent West Nile Virus Infections in Horses and Human West Nile Cases

A total of 37 recent infections in horses were confirmed during the study period, with 18 in eastern Croatia and 19 in central Croatia (Table 2). Recent WNV infections in horses were confirmed every year except in 2023, when the early detection and surveillance program for WND did not include IgM testing in horses.

**Figure 3:**
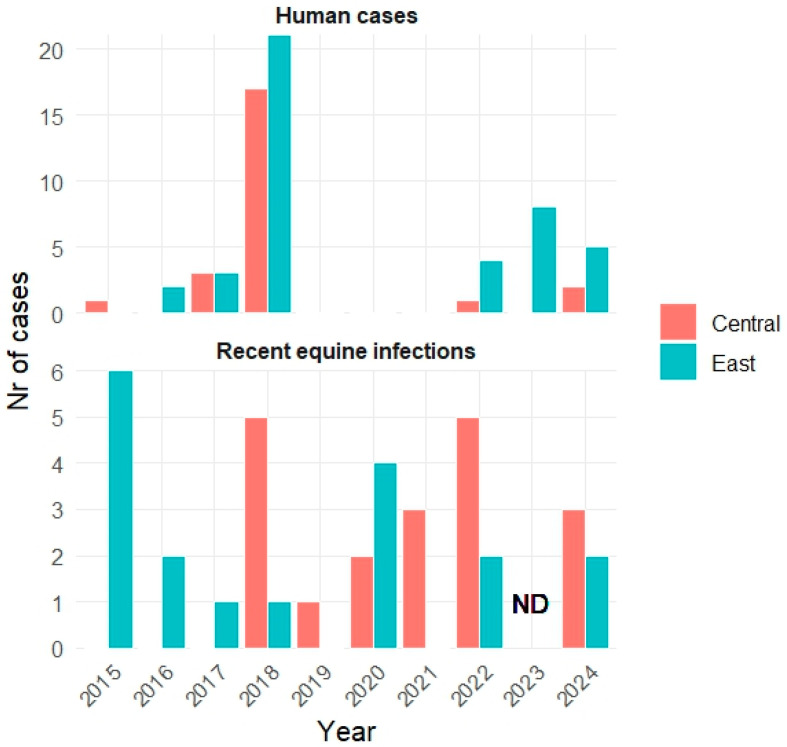
Annual numbers of human West Nile cases and recent West Nile infections in horses in Croatia (2015–2024).

In eastern Croatia, recent infections in horses were confirmed every year except in 2019 and 2021, while in central Croatia, they were confirmed continuously from the first cases in 2018 (Table 2, Figure 3).

### 3.4. Association of Equine West Nile Virus IgG Seroprevalence with Recent Equine Infections and Human West Nile Cases

The association between equine WNV IgG prevalence at specific locations and recent equine infections or human WNV cases during the same transmission season was analyzed. The results confirmed, at the location level, that seroprevalence was significantly higher (*p* = 0.049) in areas where recent equine infections had been confirmed, whereas this was not demonstrated for human WNV cases. When the studied locations were grouped into Central and Eastern Croatia, higher IgG prevalence was observed in years with confirmed recent equine infections in Central Croatia (*p* = 0.02). In contrast, this association was not confirmed for Eastern Croatia, nor was an association between IgG prevalence in horses and human WNV cases during the same transmission season found (Table 3).

Analysis of human cases and recent equine infections as predictors of WNV IgG seroprevalence in horses showed that the number of cases was not significantly associated with equine WNV IgG seroprevalence in the same transmission season. However, each confirmed human WNV case was associated with 4% higher odds of increased equine WNV IgG seroprevalence in the following season, while each additional recent equine infection was associated with 7% higher odds (Table 4).

In contrast, WNV IgG seroprevalence in horses was not a significant predictor of recent infections in horses during either the same transmission season (IRR per 10% increase in seropositivity, 0.90; 95% CI, 0.62–1.28; *p* = 0.55) or the subsequent transmission season (IRR per 10% increase in seropositivity, 0.95; 95% CI, 0.72–1.25; *p* = 0.73). It was also not confirmed as a significant predictor of WNV cases in humans in either the same season (IRR per 10% increase in seropositivity, 1.09; 95% CI, 0.52–2.14; *p* = 0.77) or the following season (IRR per 10% increase in seropositivity, 1.36; 95% CI, 0.65–2.78; *p* = 0.32).

### 3.5. Impact of Monthly Temperature Increase on the Risk Ratio of Human West Nile Cases and Recent Infections in Horses

Given the confirmed significant temperature variations during the study period (Table 1) and the seasonal characteristics of the disease, the impact of monthly temperature increases on the risk ratio of human WNV cases during the same transmission season was investigated (Table 5). The results confirmed that the risk ratio for human WNV cases increased significantly with rising temperature in four months. For each 1 °C increase, the risk ratio for human WNV cases was 1.678 times higher for a temperature increase in April and 1.589 times higher for an increase in August. The increase in risk ratio associated with a temperature rise in May and October was even more pronounced, with values of 2.296 and 2.290, respectively (Table 5).

The opposite effect was confirmed for February, when a 1 °C increase in temperature was associated with a significant reduction in the risk ratio of human WNV cases during the same transmission season (IRR 0.627; 95% CI 0.498–0.791; *p* < 0.001).

An increase in temperature in April, May, and August significantly increased the risk ratio of human WNV cases in Central Croatia (IRR 2.329, 95% CI 1.618–3.352, *p* < 0.001; IRR 4.129, 95% CI 2.476–6.885, *p* < 0.001; IRR 3.099, 95% CI 1.424–6.745, *p* = 0.013; respectively), whereas an increase in temperature in February decreased the risk ratio (IRR 0.576, 95% CI 0.407–0.816, *p* = 0.008). In East Croatia, only an increase in temperature in May significantly increased the risk ratio of human WNV cases (IRR 2.094, 95% CI 1.307–3.354, *p* = 0.025).

Because the epidemic year 2018 differed significantly in the number of recorded human WNV cases, the impact of monthly temperature increases on the number of human WNV cases was also analyzed with the 2018 data excluded. The results confirmed that, even without the epidemic year, a 1 °C increase in May increased the risk ratio of human WNV cases by 1.758 times, while a temperature increase in July had a similar effect, with the risk ratio increasing by 1.741 times in these non-epidemic years (Table 5).

Additionally, the relationship between temperature and human cases was assessed using GAMs, where a significant non-linear association was found (edf = 7.306, *p* < 0.001; Figure S1). When checking for month-specific influences, the temperature was significantly associated with human cases in most months, with both linear and non-linear relationships observed. Specifically, May and July stood out, with May having a linear (edf = 1.000, *p* < 0.001) and July a non-linear (edf = 3.952, *p* < 0.001; Figure S2, Table S1) relationship, confirming the two months that were significant in the month analyses without the epidemic year 2018. In July, the relationship between temperature and human cases exhibited a peak at approximately 22.12 °C, suggesting an optimal temperature range for transmission. Beyond this temperature, the effect plateaued or declined, indicating reduced transmission efficiency at higher temperatures.

A statistically significant effect of monthly temperature deviations on the occurrence of recent WNV infections in horses during the same transmission season was not confirmed for any month.

#### Comparison of Pre-Specified Candidate Models

To reduce the risk of overfitting, after the monthly temperature analyses and GAMs, we restricted the analysis to a limited set of candidate models pre-specified based on biological plausibility and initial analyses, including individual months and selected multi-month combinations (April, May, July, April–May, May–July, and April–May–July). Based on AIC, the best model was the one using only the month of May as a predictor. A full comparison of candidate models is provided in Supplementary Table S2.

### 3.6. Monthly Temperature Deviations During the Epidemic Year 2018

**Figure 4:**
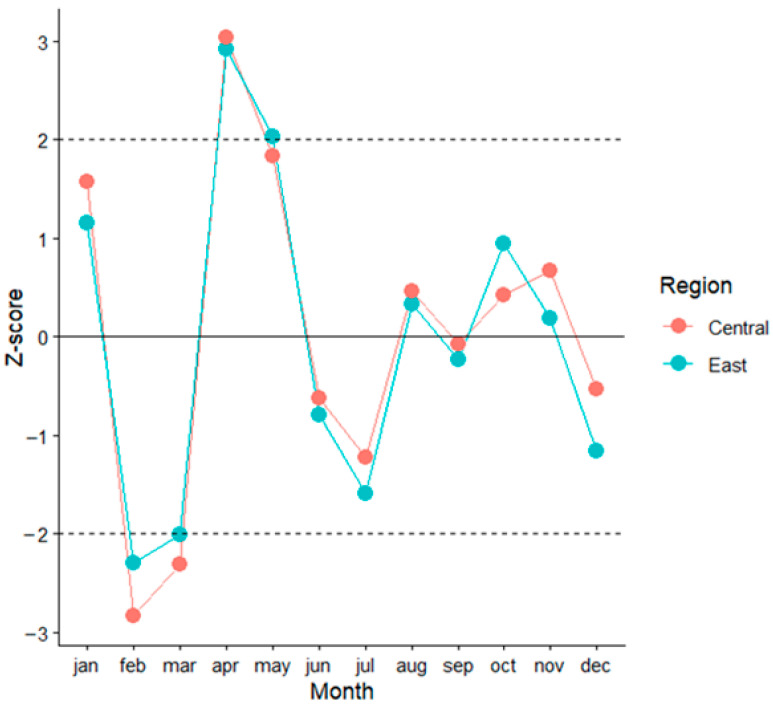
Monthly temperature deviations during the epidemic year 2018 from the study period (2015–2024) average.

Given the epidemic characteristics, monthly temperature deviations during 2018 were specifically analyzed in relation to the ten-year average for the study period. The results confirmed that, in continental Croatia, significant deviations were recorded as lower temperatures in February (z-score = −2.588) and March (z-score= −2.146), and higher temperatures in April, with a marked deviation (z-score = 3.021), as well as in May (z-score = 1.936). The same temperature pattern was confirmed at the regional level in both regions. In Central Croatia, temperatures were significantly lower in February (z-score = −2.833) and March (z-score = −2.307), and higher in April (z-score = 3.034) and May (z-score = 1.831). In Eastern Croatia, February (z-score= −2.293) and March (z-score= −2.008) were significantly colder, while April (z-score = 2.920) and May (z-score = 2.029) were warmer. For the remaining months, the differences in continental Croatia and at the regional level were less pronounced (Figure 4).

### 3.7. Evaluation of Monthly Temperature-Based Predictive Models for Human West Nile Cases

To identify the best predictive model for human WNV cases, we evaluated models using various combinations of monthly temperatures of April, May, and July, including individual months and combined multi-month temperature patterns. Among these, the model including May temperatures demonstrated the best overall predictive performance. It had the lowest prediction error (RMSE = 12.9; MAE = 7.02) and the highest Spearman correlation between observed and predicted values (ρ = 0.63).

A full comparison of predictive models and observed versus predicted values by year is provided in Supplementary Tables S3 and S4, and Figure S3.

## 4. Discussion

Reflecting WNV epidemiology, numerous studies have highlighted the importance of climatic factors in determining vector abundance, which directly influences the risk of human and equine WNV cases in endemic areas. Most studies have investigated the effects of temperature, precipitation, and humidity [19,20,21]. Accordingly, over the ten-year study period, our study analyzed the same parameters and identified statistically significant temperature deviations, with an annual increase of 0.16 °C in continental Croatia. A comparable value was observed in Central and Eastern Croatia, where a similar upward trend was evident. This result is consistent with the observed warming trend in Croatia, which is more pronounced in the continental than in the coastal region [22], whereas the slightly higher values observed in our study may be explained by the shorter study period. Precipitation and relative humidity did not show clear trends in deviation. Given that previous studies have reported the strong association between temperature and WNV infections [20], while the effect of precipitation appears more variable [23], our analysis primarily focused on the influence of temperature on WNV infections.

The WNV surveillance program in Croatia has been continuously implemented through the determination of IgG prevalence in sentinel horses since 2011. During the study period, prevalence in Eastern Croatia, the area where the first human cases were recorded, was consistently significantly higher than in Central Croatia. However, the increasing trend was almost identical in both regions, suggesting that its dynamics may be influenced by similar abiotic factors, as also confirmed for temperature in the study areas.

Analysis of the association between IgG prevalence in horses, recent equine infections, and WNV human cases showed that prevalence was significantly higher in areas where recent horse infections were detected in the same season, while no spatial association was found with human cases. At the regional level, this pattern was also observed in Central Croatia, but not in Eastern Croatia. This difference may result from variations in prevalence between these regions. In highly endemic areas with high baseline prevalence from the previous season, such as Eastern Croatia, no significant further increase in prevalence is observed during seasons in which surveillance confirms recent horse infections, whereas such an increase is observed in regions with lower baseline prevalence. The lack of a clear spatial association between increased IgG prevalence in horses and human WNV infections may be explained by the greater exposure of horses compared to humans. This is further supported by evidence that human WNV cases tend to appear later in the transmission season and may be preceded by infections in horses [24]. Another possible explanation is that horse surveillance is conducted from June to October, whereas human WNV cases most commonly occur from August to October.

Recent equine infections and human WNV cases have been recorded at various locations; however, no direct local-level association during the transmission season has been demonstrated, nor has predictive value for the subsequent season been confirmed, likely due to the relatively small number of cases.

Because the surveillance program using horses as sentinel animals runs from June to October, whereas human WNV cases are usually recorded only from August to October, predictions of increased local prevalence based on recent equine infection or human WNV cases are not confirmed within the same season but become evident in the following season. This may be explained by the fact that IgG antibodies in horses remain detectable for more than one year [25].

Previous study has shown that temperature plays a central role in the WNV epidemiology, influencing vector biology and seasonal activity, viral replication within the mosquitoes, and the processes associated with the maintenance and termination of diapause in *Culex pipiens* [19,26]. Analysis of monthly temperatures during the study period showed the strongest effect on the risk ratio of human WNV cases with increasing temperatures in May and October, and a somewhat weaker effect in April and August. This may be related to rising temperatures at the beginning of the season, in April and May, which may support the development of a substantial vector population, including infected individuals. In addition, higher temperatures in August may contribute to further population growth, while elevated temperatures in October may extend the transmission season. Together, these factors are likely to have a significant effect on the level of viral activity and, consequently, on the number of human WNV cases.

Although direct data on the effects of a warm winter followed by a subsequent cold spell on mosquito populations remain limited, available research suggests that unstable winter temperatures may negatively affect overwintering *Culex pipiens* populations. Warmer conditions increase metabolic expenditure and may promote earlier activation, whereas subsequent exposure to cold may reduce survival [27,28,29]. In our study, an increase in temperature in February was associated with a decrease in the risk ratio of human WNV cases in the following season. A possible hypothesis is that premature termination of diapause in female mosquitoes, followed by subsequent cold spells that reduce their survival, ultimately affected vector abundance during the peak transmission season.

To further investigate the effect on the risk ratio of human WNV cases, an analysis was performed excluding data from the epidemic year 2018 to remove its influence on the results. The findings confirmed a significant effect of increased temperatures in May, with an additional effect of elevated temperatures in July, corresponding to the seasonal peak.

Due to the specificity of the epidemic year 2018, which had the highest number of human WNV cases in Croatia and across Europe, monthly temperature deviations during that year were compared to the average for the research period. In continental Croatia, as well as by region, it was confirmed that in 2018, extremely high temperatures in April and May were preceded by significantly lower temperatures in February and March. From these and previously discussed monthly deviations, it follows that for a significantly higher number of cases in the season, temperatures in the preceding transmission season are also important, as they do not cause premature exit from diapause and favor vector activity at the beginning of the season.

Based on these findings, the most informative prediction model for human WNV cases during the transmission season was identified through the analysis of individual and combined average monthly temperatures. Monitoring average temperatures in April and May emerged as the most reliable predictor of the intensity of the upcoming WNV season in humans, with additional analyses confirming increased May temperatures as the most consistent predictor. These results may provide valuable guidance for public health planning, including the timely implementation of control measures and preparedness of the healthcare system. Furthermore, the observed relationship between July temperature and human WNV cases suggests the presence of an optimal transmission threshold around 22.12 °C, highlighting the importance of temperature dynamics and warranting further investigation to more fully elucidate the role of abiotic factors in shaping WNV epidemiology.

There are some limitations of this study that need to be addressed. Since confirmatory VNT was not performed, there is a possibility of cross-reactive antibodies with other flaviviruses that circulate in the same areas. However, any potential low level of cross-reactivity would likely have remained comparable throughout the study period, since surveillance was conducted in the same geographic areas and using the same diagnostic method across all years. In addition, due to the relatively small number of human WNV cases and recent equine infections, as well as the absence of monitoring of recent equine infections in 2023, the results of this study should be further validated over a longer period and across different geographic regions.

## 5. Conclusions

WNV is a typical emerging vector-borne zoonosis whose surveillance requires a multidisciplinary approach consistent with the One Health framework. The significant warming observed during the study period, along with increasing seroprevalence, suggests that these trends are likely to persist, posing an increasingly significant challenge to public health. In addition to the value of data obtained by testing horses as sentinel animals through annual prevalence trends, these findings indicate that prevalence data provide clear insight into viral activity even when human disease is not reported. Furthermore, beyond the established role of detecting recent equine infections as an early indicator of the transmission season and a warning signal for human cases, monitoring climatic factors, particularly temperature, may serve as a candidate predictor of the potential intensity of the upcoming WNV season and inform public health preparedness efforts. This study confirms that increased May temperatures are the most consistent predictor of the intensity of the upcoming WNV transmission season, providing a potentially valuable tool for public health planning, while the results for July suggest the presence of an optimal transmission temperature at the peak of the season. In addition, the identified specific temperature pattern of the epidemic year, characterized by below-average temperatures in February and March followed by above-average temperatures in April and May, warrants further investigation to improve predictive models for future seasons. Continued research across a broader range of areas and over longer time series, along with the examination of additional abiotic factors, other animal species, and vectors, is needed to fully understand the occurrence patterns of this zoonosis.

## Figures and Tables

**Table 1:** Temporal trends in annual temperature, precipitation, and humidity in continental Croatia, Eastern Croatia, and Central Croatia during 2015–2024, expressed as annual change and Spearman’s correlation coefficients.

Region	Temperature				Precipitation				Humidity			
	°C/Year	*p*	rho (ρ)	*p*	mm/Year	*p*	rho (ρ)	*p*	%/Year	*p*	rho (ρ)	*p*
Continental	0.16	0.02	0.67	0.03	1.09	0.90	−0.05	0.88	−0.07	0.63	−0.21	0.57
Eastern	0.16	0.02	0.61	0.06	−2.49	0.80	−0.15	0.68	−0.24	0.098	−0.54	0.11
Central	0.16	0.02	0.66	0.04	10.79	0.43	0.21	0.56	0.10	0.53	0.16	0.66

**Table 2:** West Nile virus IgG prevalence in horses, confirmed horse recent infection (IgM positive), and human cases by year and regions.

Year	Region	Seroprevalence in Horses					Human Cases
		N Tested	WNV IgG N (%)	95% CI	WNV IgM N (%)	95%CI	
2015	Eastern	399	97 (24.31)	20.36–28.75	6 (1.50)	0.55–3.24	0
	Central	357	16 (4.48)	2.78–7.16	0 (0.00)	NA	1
	Continental	756	113 (14.95)	12.58–17.67	6 (0.79)	0.29–1.72	1
2016	Eastern	448	98 (21.88)	18.29–25.93	2 (0.45)	0.05–1.60	2
	Central	326	22 (6.75)	4.50–10.01	0 (0.00)	NA	0
	Continental	774	120 (15.50)	13.12–18.22	2 (0.26)	0.03–0.93	2
2017	Eastern	445	102 (22.92)	19.26–27.05	1 (0.22)	0.01–1.25	3
	Central	402	41 (10.20)	7.61–13.54	0 (0.00)	NA	3
	Continental	847	143 (16.88)	14.51–19.55	1 (0.12)	0.00–0.66	6
2018	Eastern	462	99 (21.43)	17.93–25.40	1 (0.22)	0.01–1.20	21
	Central	395	31 (7.85)	5.58–10.92	5 (1.27)	0.41–2.93	17
	Continental	857	130 (15.17)	12.92–17.73	6 (0.70)	0.26–1.52	38
2019	Eastern	116	56 (48.28)	39.38–57.28	0 (0.00)	NA	0
	Central	147	26 (17.69)	12.37–24.65	1 (0.68)	0.02–3.73	0
	Continental	263	82 (31.19)	25.89–37.01	1 (0.38)	0.01–2.10	0
2020	Eastern	158	60 (37.97)	30.78–45.74	4 (2.53)	0.69–6.35	0
	Central	99	15 (15.15)	9.40–23.50	2 (2.02)	0.25–7.11	0
	Continental	257	75 (29.18)	23.96–35.01	6 (2.33)	0.86–5.01	0
2021	Eastern	136	57 (41.91)	33.95–50.31	0 (0.00)	NA	0
	Central	180	33 (18.33)	13.36–24.63	3 (1.67)	0.34–4.79	0
	Continental	316	90 (28.48)	23.79–33.69	3 (0.95)	0.20–2.75	0
2022	Eastern	203	88 (43.35)	36.72–50.23	2 (0.99)	0.12–3.51	4
	Central	145	21 (14.48)	9.67–21.13	5 (3.45)	1.13–7.86	1
	Continental	348	109 (31.32)	26.68–36.38	7 (2.01)	0.81–4.10	5
2023	Eastern	25	15 (60.00)	40.74–76.6	ND	NA	8
	Central	68	10 (14.71)	8.19–25.00	ND	NA	0
	Continental	93	25 (26.88)	18.92–36.68	ND	NA	8
2024	Eastern	255	131 (51.37)	45.26–57.44	2 (0.78)	0.10–2.80	5
	Central	220	41 (18.64)	14.05–25.30	3 (1.36)	0.28–3.93	2
	Continental	475	172 (36.21)	32.02–40.63	5 (1.05)	0.34–2.44	7
2015–2024	Eastern	2647	803 (30.34)	28.61–32.11	18 (0.68)	0.40–1.07	43
	Central	2339	256 (10.94)	9.74–12.27	19 (0.81)	0.49–1.27	24
	Continental	4986	1059 (21.24)	20.11–22.40	37 (0.74)	0.52–1.02	67

CI = Confidence interval, ND = Not determined (IgM testing in horses was not performed due to changes in the early detection and surveillance program for West Nile disease in the Republic of Croatia in 2023), NA = Not applicable.

**Table 3:** Association of equine WNV IgG seroprevalence with human WNV cases and recent equine WNV infections at the location and regional levels, 2015–2024.

Level of Analysis	Region	Indicator	N Locations in Transmission Seasons		Equine WNV IgG Prevalence				*p* MWU
					Median		Mean		
			Positive **	Negative	Positive	Negative	Positive	Negative	
Location level	–	Human cases	14	26	20.86	20.80	24.78	25.09	0.92
	–	Equine * infections	19	17	23.23	13.85	27.66	19.71	0.049
Regional level	Eastern	Human cases	8	12	25.48	38.68	35.27	37.69	0.62
	Central		6	14	10.97	12.89	10.85	14.28	0.43
	Eastern	Equine infections	12	6	34.82	39.02	33.8	37.85	0.54
	Central		7	11	17.72	8.61	17.13	9.82	0.02

Locational level = all 4 locations combined; * Recent infections; ** Positive locations = locations with detected human WNV cases or recent equine WNV infections, MWU = Mann–Whitney U test.

**Table 4:** Association of human WNV cases and recent equine WNV infections with equine WNV IgG seroprevalence in the same and subsequent transmission season at the regional level.

Predictor	Transmission Season	Model	N	OR per +1 Case	95% CI	*p*
Human WNV cases	same	year	20	0.99	0.98–1.00	0.054
	following	year	18	1.04	1.03–1.06	<0.001
Equine recent infections	same	year	18	1.00	0.96–1.04	0.97
	following	year	16	1.07	1.02–1.11	<0.01

OR = Odds ratio; CI = Confidence interval.

**Table 5:** Impact of monthly temperature increases on the risk ratios for human West Nile cases.

Month	2015–2024		2015–2024 (Excluding 2018)	
	IRR (95% CI)	*p* *	IRR (95% CI)	*p* *
January	1.568 (0.950–2.587)	0.135	0.882 (0.597–1.304)	0.869
February	0.627 (0.498–0.791)	<0.001	0.985 (0.657–1.476)	0.942
March	0.649 (0.406–1.037)	0.135	1.277 (0.883–1.848)	0.465
April	1.678 (1.349–2.088)	<0.001	1.095 (0.777–1.544)	0.869
May	2.296 (1.800–2.929)	<0.001	1.758 (1.162–2.658)	0.045
June	0.828 (0.521–1.318)	0.512	1.133 (0.624–2.056)	0.869
July	0.716 (0.345–1.489)	0.495	1.741 (1.162–2.608)	0.045
August	1.589 (1.052–2.401)	0.067	1.343 (0.907–1.989)	0.424
September	1.112 (0.768–1.608)	0.575	1.127 (0.599–2.121)	0.869
October	2.290 (1.426–3.676)	0.002	1.373 (0.927–2.033)	0.424
November	1.136 (0.769–1.677)	0.570	1.066 (0.658–1.726)	0.869
December	0.793 (0.549–1.144)	0.322	1.081 (0.646–1.808)	0.869

IRR = incidence rate ratio; CI = Confidence interval; * *p*-values were adjusted using the Benjamini–Hochberg false discovery rate (FDR) method.

## Data Availability

The original contributions presented in this study are included in the article/Supplementary Materials. Further inquiries can be directed to the corresponding authors.

## Supplementary Materials

The following supporting information can be downloaded at: https://www.mdpi.com/article/10.3390/pathogens15050509/s1, Figure S1: Generalized Additive Model (GAM) analyses. Temperature showed a significant non-linear association with human cases (edf = 7.306, *p* < 0.001). The smooth function indicates a non-linear association between temperature and human cases. The risk decreases at moderate temperatures (around 8–10°C), followed by a marked increase at higher temperatures, suggesting elevated transmission potential in warmer conditions. Shaded (dashed) lines represent 95% confidence intervals, and tick marks along the x-axis indicate the distribution of observations; Figure S2: Month-specific smooth functions showing the association between temperature and human cases across months. Dashed lines represent 95% confidence intervals, and tick marks along the x-axis indicate the distribution of observations; Figure S3: Observed and predicted human WNV cases by year for the May temperature model, obtained using forward-chaining validation; Table S1: Effective degrees of freedom (edf) and *p*-values for month-specific smooth terms of temperature from the generalized additive model (GAM); Table S2: Comparison of a limited set of pre-specified candidate models; Table S3: Comparison of predictive performance for pre-specified temperature-based models using forward-chaining validation. Model performance is summarized by root mean square error (RMSE), mean absolute error (MAE), and Spearman’s rank correlation (ρ) between observed and predicted human WNV cases; Table S4: Observed and predicted human WNV cases by year for the May temperature model, obtained using forward-chaining validation. Each year was predicted using only the preceding years.

## Abbreviations

The following abbreviations are used in this manuscript:

WNV	West Nile virus
ELISA	Enzyme-linked immunosorbent assay
ECDC	European Centre for Disease Prevention and Control
CSF	Cerebrospinal fluid
CI	Confidence interval
OR	Odds ratio
IRR	Incidence rate ratio
