# Supplementary material for: Temperature-Based Predictions for West Nile Virus Outbreaks in Endemic Regions of Continental Croatia
Source: Pathogens. 2026 May 8;15(5):509. doi: 10.3390/pathogens15050509 (PMC13209223; doi:10.3390/pathogens15050509)
Supplement: Supplementary file 1 [file pathogens-15-00509-s001.zip › pathogens-4275705-supplementary.pdf]

Figure S1. Generalized Additive Model (GAM) analyses. Temperature showed a significant non-linear association with human cases (edf = 7.306, p<0.001). The smooth function indicates a non-linear association between temperature and human cases. The risk decreases at moderate temperatures (around 8–10°C), followed by a marked increase at higher temperatures, suggesting elevated transmission potential in warmer conditions. Shaded (dashed) lines represent 95% confidence intervals, and tick marks along the x-axis indicate the distribution of observations.

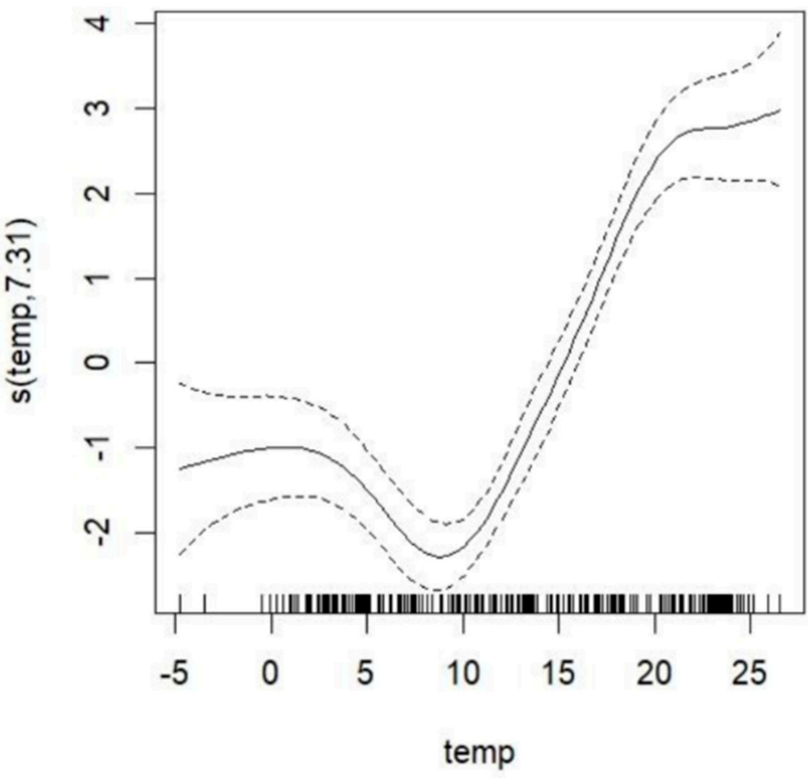

Figure S2. Month-specific smooth functions showing the association between temperature and human cases across months. Dashed lines represent 95% confidence intervals, and tick marks along the x-axis indicate the distribution of observations.

Allowing month-specific smooth terms significantly improved model fit (ΔAIC > 500, $p<0.001$) compared with a model assuming a common temperature effect across months (Figure S1), indicating that the effect of temperature varies across months. Temperature was significantly associated with human cases in most months, with both linear and non-linear relationships observed. The strength and shape of the association differed across months, with a linear increase in May (edf = 1.000, $p<0.001$), and a pronounced peak in July (edf = 3.952, $p<0.001$). In July, the relationship between temperature and human cases exhibited a peak at approximately 22.12°C, suggesting an optimal temperature range for transmission. Beyond this temperature, the effect plateaued or declined, indicating reduced transmission efficiency at higher temperatures.

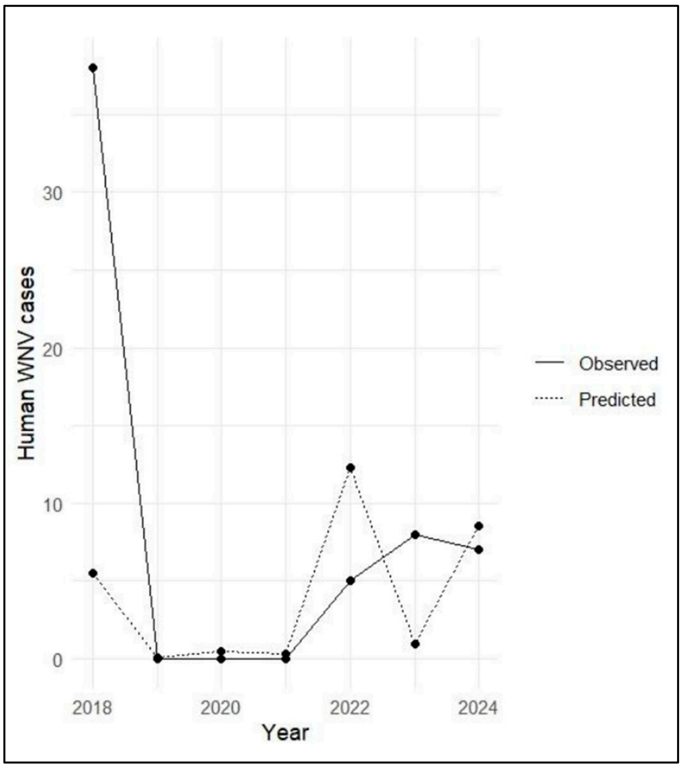

Figure S3. Observed and predicted human WNV cases by year for the May temperature model, obtained using forward-chaining validation.

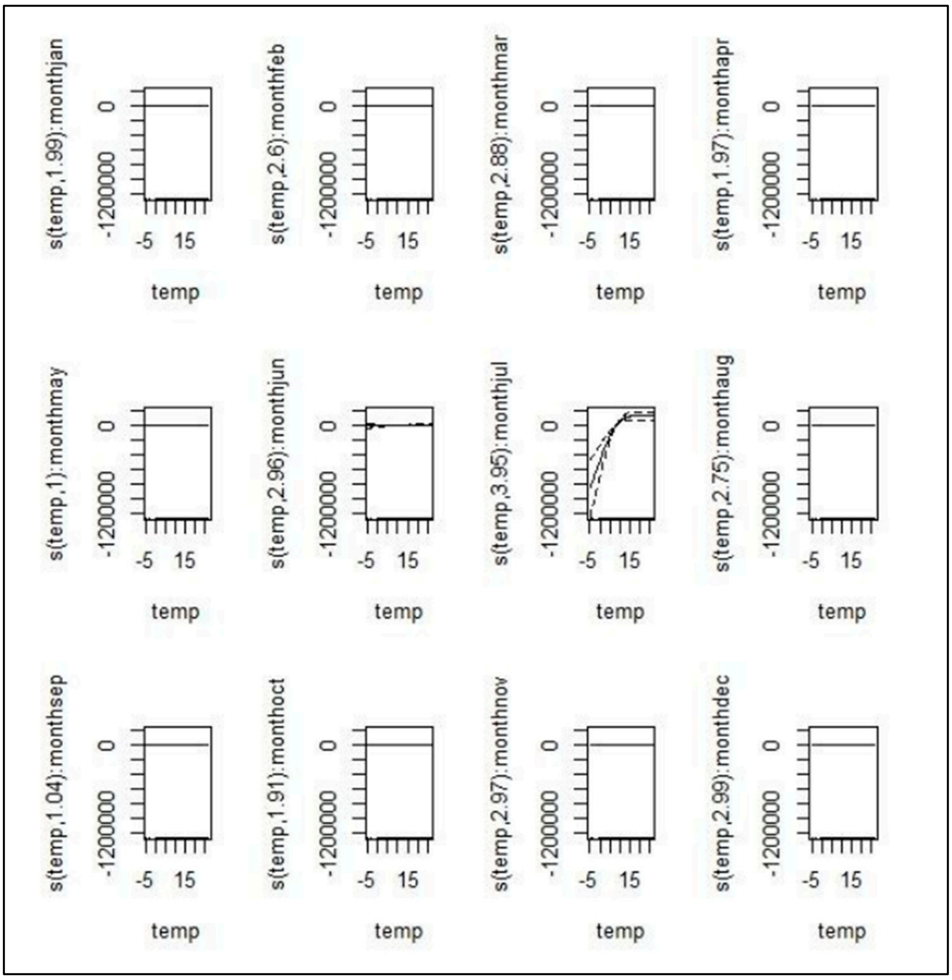

Table S1. Effective degrees of freedom (edf) and p-values for month-specific smooth terms of temperature from the generalized additive model (GAM).

**Month**	**edf**	***p*-value**
January	1.988	**<0.001**
February	2.603	**<0.001**
March	2.879	**<0.001**
April	1.971	**<0.001**
May	1.000	**<0.001**
June	2.959	**<0.001**
July	3.952	**<0.001**
August	2.752	**0.002**
September	1.043	0.602
October	1.906	**<0.001**
November	2.967	**0.025**
December	2.987	**<0.001**

edf = effective degrees of freedom. Statistically significant *p*-values ($p<0.05$) are shown in bold.

Table S2. Comparison of a limited set of pre-specified candidate models.

Model/Predictor (Temperature, °C)	IRR per +1 °C (95% CI)	*p*-value	AIC	N	FDR-adjusted *p*
April	1.458 (1.149–1.850)	**0.002**	123.091	20	**0.004**
May	1.935 (1.454–2.574)	**<0.001**	106.821	20	**<0.001**
July	0.605 (0.354–1.033)	0.066	148.172	20	0.079
April–May	1.670 (1.315–2.120)	**<0.001**	111.280	20	**<0.001**
May–July	1.780 (0.934–3.389)	0.080	147.375	20	0.080
April–May–July	2.072 (1.249–3.438)	**0.005**	124.147	20	**0.007**

IRR = incidence rate ratio; CI = confidence interval; AIC = Akaike information criterion; FDR = false discovery rate. IRR represents the change per +1 °C increase in temperature. Lower AIC values indicate better model fit. Statistically significant *p*-values ($p<0.05$) are shown in bold.

Table S3. Comparison of predictive performance for pre-specified temperature-based models using forward-chaining validation. Model performance is summarized by root mean square error (RMSE), mean absolute error (MAE), and Spearman's rank correlation (ϱ) between observed and predicted human WNV cases

Model/Predictor (Temperature, °C)	RMSE	MAE	Spearman's ϱ
May	12.9	7.02	0.630
April–May	14.7	7.79	-0.074
Apr	14.9	8.66	-0.296
April–May–July	17.4	10.5	0.074
May–July	20.6	14.3	0.593
July	23.4	16.1	-0.222
April–July	31.6	18.0	-0.148

RMSE = root mean square error; MAE = mean absolute error; ϱ = Spearman's rank correlation coefficient. Lower RMSE and MAE indicate better predictive performance, while higher ϱ indicates stronger monotonic agreement between observed and predicted values.

Table S4. Observed and predicted human WNV cases by year for the May temperature model, obtained using forward-chaining validation. Each year was predicted using only the preceding years.

Model/Predictor (Temperature, °C)	Year	Observed cases	Predicted cases
May	2018	38	5.528
May	2019	0	0.082
May	2020	0	0.437
May	2021	0	0.272
May	2022	5	12.308
May	2023	8	0.934
May	2024	7	8.513
